# The Maternal Donor of *Chrysanthemum* Cultivars Revealed by Comparative Analysis of the Chloroplast Genome

**DOI:** 10.3389/fpls.2022.923442

**Published:** 2022-06-02

**Authors:** Yufen Xu, Borong Liao, Kate L. Ostevik, Hougao Zhou, Fenglan Wang, Baosheng Wang, Hanhan Xia

**Affiliations:** ^1^College of Horticulture and Landscape Architecture, Zhongkai University of Agriculture and Engineering, Guangzhou, China; ^2^Coconut Research Institute of Chinese Academy of Tropical Agricultural Sciences, Wenchang, China; ^3^Department of Evolution, Ecology and Organismal Biology, University of California, Riverside, CA, United States; ^4^Key Laboratory of Plant Resources Conservation and Sustainable Utilization, South China Botanical Garden, Chinese Academy of Sciences, Guangzhou, China

**Keywords:** *Chrysanthemum*, chloroplast genome, maternal donor, genetic marker, phylogenetic analysis

## Abstract

Chrysanthemum (*Chrysanthemum morifolium* Ramat) is an important floricultural crop and medicinal herb. Modern chrysanthemum cultivars have complex genetic backgrounds because of multiple cycles of hybridization, polyploidization, and prolonged cultivation. Understanding the genetic background and hybrid origin of modern chrysanthemum cultivars can provide pivotal information for chrysanthemum genetic improvement and breeding. By now, the origin of cultivated chrysanthemums remains unclear. In this study, 36 common chrysanthemum cultivars from across the world and multiple wild relatives were studied to identify the maternal donor of modern chrysanthemum. Chloroplast (cp) genomes of chrysanthemum cultivars were assembled and compared with those of the wild relatives. The structure of cp genomes was highly conserved among cultivars and wild relatives. Phylogenetic analyses based on the assembled cp genomes showed that all chrysanthemum cultivars grouped together and shared 64 substitutions that were distinct from those of their wild relatives. These results indicated that a diverged lineage of the genus *Chrysanthemum*, which was most likely an extinct or un-sampled species/population, provided a maternal source for modern cultivars. These findings provide important insights into the origin of chrysanthemum cultivars, and a source of valuable genetic markers for chrysanthemum breeding programs.

## Introduction

Chrysanthemum (*Chrysanthemum morifolium* Ramat) is one of the most popular and economically important floricultural crops in the world, noted for its ornamental, nutritional, and medicinal values (Shih et al., 2011; Shinoyama et al., 2012). It was first cultivated in China approximately 1,600 years ago, then successively introduced to Japan, Europe, and North America (Chen, 1985, 2012; Shih et al., 2011). Modern chrysanthemum cultivars are mainly allohexaploids (2*n* = 6*x* = 54; Kumari et al., 2019). They have diverse floral morphologies, lignified stems, and can thrive in a wide range of habitats (e.g., urban, rural and farmland). The complex genetic and phenotypic variation of *C. morifolium* is thought to be the result of multiple cycles of hybridization, polyploidization, and artificial selection (Shih et al., 2011; Chen, 2012). Interspecific hybridization among modern chrysanthemums and their wild relatives, are still widely used for cultivar improvement (Kumari et al., 2019). The selection of parent species is a critical step for hybrid breeding. Currently, phenotypic traits are frequently used to guide the choice of germplasm to develop new chrysanthemum cultivars with novel appearances and improved stress tolerance (Kumari et al., 2019). However, hybrid progeny can exhibit extreme or “transgressive” traits relative to their progenitors (Rieseberg et al., 1999; Schwarzbach et al., 2001). Thus, hybridization between parent species selected according to phenotype may not produce offspring with the expected characters, e.g., a combination of the parents’ phenotypic characters. This may be an especially common problem for chrysanthemum, because *C. morifolium* cultivars and wild relatives have complex genetic backgrounds because of high ploidy levels and a long history of hybridization and artificial selection. Understanding the genetic background and hybrid origin of chrysanthemum cultivars can provide pivotal information for choosing germplasm resources, reduce the time-consuming process of artificial crossing, and ultimately facilitate genetic improvement and breeding programs (Lim et al., 2008; Kuligowska et al., 2016).

The origin of cultivated chrysanthemums has attracted great attention over the past decades. Previous studies based on morphological and genetic data show that cultivated chrysanthemums are derived from interspecific hybridization and polyploidization, involving *Chrysanthemum indicum* L., *Chrysanthemum zawadskii* Herbich, *Chrysanthemum argyrophyllum* Y. Ling, *Chrysanthemum dichrum* (C. Shih) H. Ohashi & Yonek, *Chrysanthemum nankingense* (Hand.-Mazz.) X.D. Cui, and *C. vestitum* (Hemsl.) Stapf (Chen, 1985, 2012; Dai et al., 1998, 2005; Fukai et al., 2003; Ma et al., 2020). Multiple lines of evidence point to *C. indicum* (2*n* = 4*x* = 36) and *C. vestitum* (2*n* = 6*x* = 54) as pivotal players in the origin of modern chrysanthemums. First, hybrids between *C. indicum* and *C. vestitum* show some phenotypic characters similar to modern chrysanthemums (Chen, 1985, 2012). Second, *C. indicum* is widely used for multiple purposes in Central China, where cultivated chrysanthemums most likely originated (Chen, 1985, 2012). Recently, cpDNA data has been used to investigate the origin of modern chrysanthemums (Ma et al., 2020; Qi et al., 2021). For example, Ma et al. (2020) investigated the phylogenetic relationships between chrysanthemum cultivars and wild relatives using cp genomes. They found that chrysanthemum cultivars formed a strongly supported clade on cpDNA tree and diverged from all wild species, indicating that the maternally inherited cp genome of modern chrysanthemums might be derived from an extinct progenitor (Ma et al., 2020). By sequencing two cpDNA fragments in multiple populations of wild *Chrysanthemum* species, Qi et al. (2021) found high cpDNA variation within species and suggested that the maternal progenitor of modern chrysanthemums could be an un-sampled population of wild species. Because these studies sampled only a small number of cultivated chrysanthemums (Ma et al., 2020; Qi et al., 2021), they may not capture all cpDNA variations in cultivars. Therefore, the maternal donor of modern chrysanthemums remains poorly understood.

In this study, we performed comprehensive sampling in cultivars of *C. morifolium*. A total of 36 accessions were collected to represent common cultivars of different countries and to represent different flower morphologies. The whole cp genomes of chrysanthemum cultivars were assembled compared to those of their wild relatives to determine the origin of cultivated chrysanthemums. Phylogenetic analyses based on the assembled cp genomes elucidated the maternal origin of chrysanthemum cultivars. This study sheds light on the origin of modern chrysanthemums, and provides valuable genetic resources for the development of new cultivars.

## Materials and Methods

### Sampling, DNA Extraction, and Sequencing

**Figure 1:**
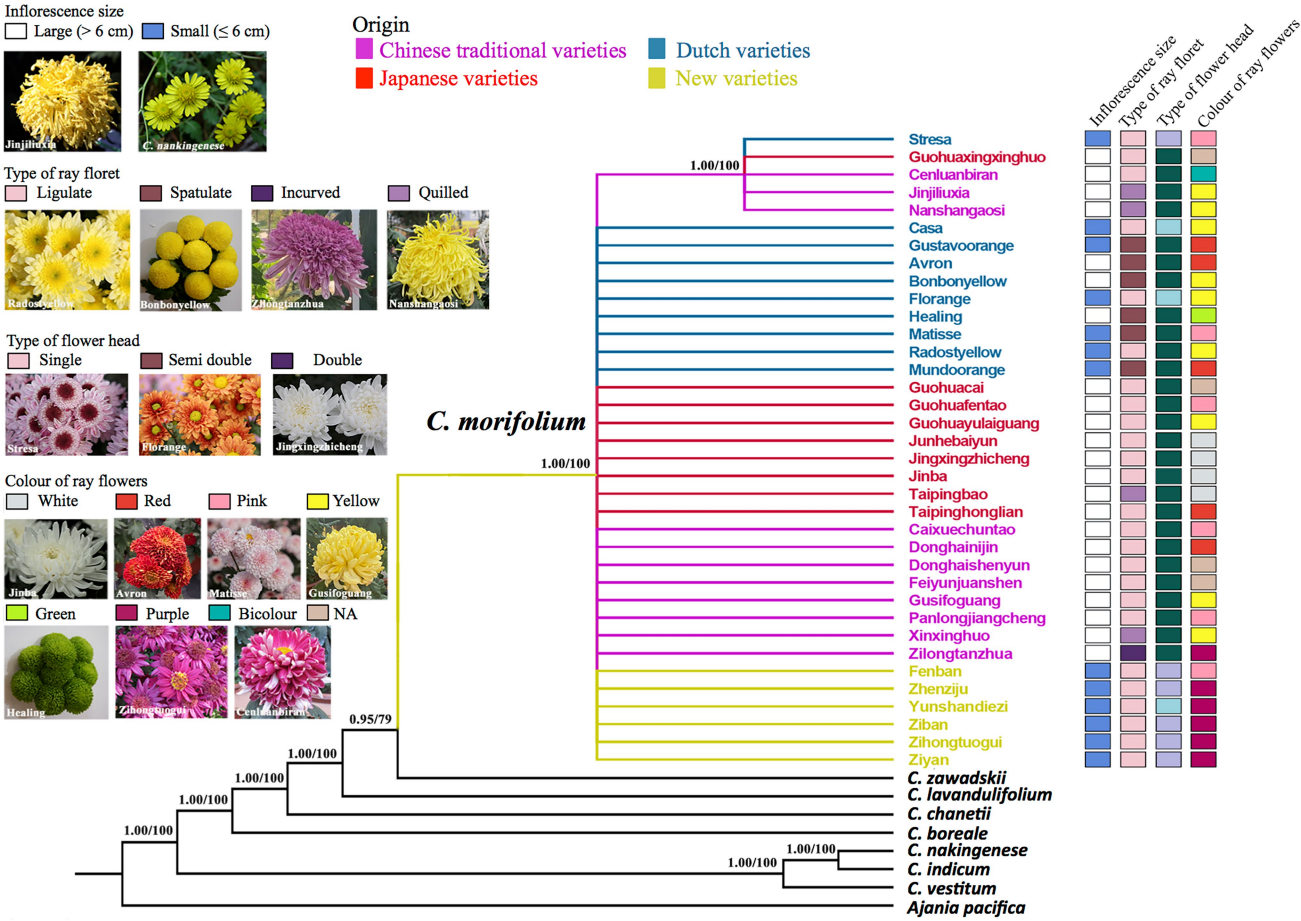
Phylogenetic relationships of chrysanthemums inferred from whole chloroplast genomes. Numbers above the branches are Bayesian posterior probability (PP) and likelihood bootsrap (BS) values. Branches with PP < 0.5 or BS < 50 are collapsed. The cultivar clades and names are indicated in different colors depending on their origin. Cultivar floral traits are showed on the right of the tree.

Thirty-six common cultivars of *C. morifolium* with different origins and flower morphologies were collected, which included 11 Chinese traditional cultivars, nine Japanese cultivars, 10 Dutch cultivars, and six cultivars (referred as HD cultivars hereafter) recently developed by Houde Agricultural Technology (Guangzhou, China). Seven wild relatives of *C. morifolium*, and an outgroup species *Ajania pacifica* (Nakai) K.Bremer & Humphries (Anthemidinae, Asteraceae) were also sampled (Table 1; Figure 1). Seedlings from Houde Agricultural Technology were obtained for all cultivars and one wild species (*Chrysanthemum nankingenese*) for genome sequencing (Table 1). For other wild and outgroup species, the cp genome sequences from GenBank were downloaded with the following accession numbers: JN867589, MW539687, MH339742, MH165287, MN883841, NC037388, NC057203 (Table 1). Details of samples were provided in Table 1.

**Table 1:** Origin and flower characteristics of the 36 chrysanthemum cultivars and seven related wild species.

Sample	Inflorescence size^a^	Type of ray floret	Type of flower head	Color of ray flowers	Voucher	Genbank accession number	Origin
*Cultivar (Chrysanthemum morifolium)*							
“Cenluanbiran”	Large	Ligulate	Double	Bicolor	ZK-CLBN-1	ON534022	China
“Caixuechuntao”	Large	Ligulate	Double	Pink	ZK-CXCT-1	ON534023	China
“Feiyunjuanshen”	Large	Ligulate	Double	NA	ZK-FYJS-1	ON534028	China
“Gusifoguang”	Large	Ligulate	Double	Yellow	ZK-GSFG-1	ON534033	China
“Donghaishenyun”	Large	Ligulate	Double	NA	ZK-DHSY-1	ON534026	China
“Panlongjiangcheng”	Large	Ligulate	Double	Pink	ZK-PLJC-1	ON534046	China
“Donghainijin”	Large	Ligulate	Double	Red	ZK-DHNJ-1	ON534025	China
“Jinjiliuxia”	Large	Quilled	Double	Yellow	ZK-JJLX-1	ON534039	China
“Nanshangaosi”	Large	Quilled	Double	Yellow	ZK-NSGS-1	ON534045	China
“Xinxinghuo”	Large	Quilled	Double	Yellow	ZK-XXH-1	ON534054	China
“Zilongtanzhua”	Large	Incurved	Double	Purple	ZK-ZLTZ-1	ON534057	China
“Taipingbao”	Large	Quilled	Double	Yellow	ZK-TPBO-1	ON534049	Japan
“Guohuacai”	Large	Ligulate	Double	NA	ZK-GHCI-1	ON534029	Japan
“Guohuaxingxinghuo”	Large	Ligulate	Double	NA	ZK-GHXX-1	ON534031	Japan
“Taipinghonglian”	Large	Ligulate	Double	Red	ZK-TPHL-1	ON534050	Japan
“Guohuafentao”	Large	Ligulate	Double	Pink	ZK-GHFT-1	ON534030	Japan
“Jingxingzhicheng”	Large	Ligulate	Double	White	ZK-JXZC-1	ON534040	Japan
“Junhebaiyun”	Large	Ligulate	Double	White	ZK-JHBY-1	ON534037	Japan
“Jinba”	Large	Ligulate	Double	White	ZK-JINB-1	ON534048	Japan
“Guohuayulaiguang”	Large	Ligulate	Double	Yellow	ZK-GYLG-1	ON534032	Japan
“Bonbonyellow”	Large	Spoon-shaped	Double	Yellow	ZK-BBYW-1	ON534036	Netherlands
“Healing”	Large	Spoon-shaped	Double	Green	ZK-HEAL-1	ON534043	Netherlands
“Avron”	Large	Spoon-shaped	Double	Red	ZK-AVRN-1	ON534035	Netherlands
“Casa”	Small	Ligulate	Semi-double	Yellow	ZK-CASA-1	ON534024	Netherlands
“Florange”	Small	Ligulate	Semi-double	Yellow	ZK-FLOR-1	ON534041	Netherlands
“Radostyellow”	Small	Ligulate	Double	Yellow	ZK-RADO-Y	ON534047	Netherlands
“Stresa”	Small	Ligulate	Single	Pink	ZK-STRE-1	ON534042	Netherlands
“Matisse”	Small	Spoon-shaped	Double	Pink	ZK-MATS-1	ON534044	Netherlands
“Gustavoorange”	Small	Spoon-shaped	Double	Red	ZK-GUST-1	ON534034	Netherlands
“Mundoorange”	Small	Spoon-shaped	Double	Red	ZK-MUND-1	ON534051	Netherlands
“Yunshandiezi”	Small	Ligulate	Semi-double	Purple	ZK-YSDZ-1	ON534053	New cultivar^b^
“Zhenziju”	Small	Ligulate	Single	Purple	ZK-ZZJU-1	ON534052	New cultivar^b^
“Ziban”	Small	Ligulate	Single	Purple	ZK-ZBAN-1	ON534055	New cultivar^b^
“Fenban”	Small	Ligulate	Single	Pink	ZK-FENB-1	ON534027	New cultivar^b^
“Zihongtuogui”	Small	Ligulate	Single	Purple	ZK-ZHTG-1	ON534056	New cultivar^b^
“Ziyan”	Small	Ligulate	Single	Purple	ZK-ZIYA-1	ON534058	New cultivar^b^
*Wild species*							
*C. boreale* (Makino) Makino	Small	Quilled	Single	Yellow	NA	NC037388	NA
*C. indicum* L.	Small	Ligulate	Single	Yellow	NA	JN867589	NA
*C. nankingense* (Hand.-Mazz.) X.D.Cui	Small	Ligulate	Single	Yellow	ZK-JHNN-1	ON534038	China
*C. zawadskii* Herbich	Small	Ligulate	Single	White, Purple, Red	NA	MW539687	NA
*C. chanetii* H. Lév.	Small	Ligulate	Single	White, Pink, Purple	NA	MH339742	NA
*C. lavandulifolium* (Fisch. ex Trautv.) Makino	Small	Ligulate	Single	Yellow	NA	MH165287	NA
*C. vestitum* (Hemsl.) Stapf	Small	Ligulate	Single	White	NA	NC057203	NA
*Outgroup*							
*Ajania pacifica* (Nakai) K.Bremer & Humphries	NA	NA	NA	NA	NA	MN883841	NA

a The inflorescence size was divided into two classes: large (>6 cm) and small (≤6 cm).

b New cultivars recently developed by Houde agricultural technology (Guangzhou, China).

NA, data not available.

Total genomic DNA from 100 mg of fresh leaves was isolated using a DNeasy Plant MiniKit (Qiagen). DNA quality was examined by electrophoresis in 1% agarose, and DNA concentration was quantified with a BioPhotometer (Eppendorf, United States). High-quality DNA samples were sent to Novogene (Beijing, China) for library construction and sequencing. Genome sequencing was conducted on the Illumina HiSeq2500 platform to obtained 150 bp pair-end reads.

### Chloroplast Genome Assembly and Annotation

*De novo* assembly of cp genomes for the 37 samples (36 cultivars and one wild species) was conducted using NOVOPlasty (Dierckxsens et al., 2017). For each sample, the cp genome was assembled by using 8–10 Gb of raw reads and *a ribulose-bisphosphate carboxylase* (*RBCL*) gene sequence from *C. indicum* (JN867589) as a seed sequence.

The cp genomes were annotated using GeSeq (Tillich et al., 2017) and Sequin was used for proofreading.^1^ The size, length of structural division, and gene content of the cp genome were measured according to the annotation results. The GC content of cp genomes was calculated using MEGA X (Kumar et al., 2018). The chloroplast genome map was obtained using OGDRAW (Greiner et al., 2019).

### Comparative Analysis of the cp Genomes

The cp genome of *C. indicum* (JN867589) was used as a reference to align and compare the *Chrysanthemum* cp genomes using mVISTA (Frazer et al., 2004), and genome collinear analysis was performed with the Mauve tool (Darling et al., 2004). The expansion and contraction of IR boundaries were analyzed using IRscope (Amiryousefi et al., 2018).

The repeat regions on cp genome were annotated using REPuter (Kurtz et al., 2001). Forward, reverse, complementary, and palindromic repeat sequences with a hamming distance of 3 and minimum repeat size of 30 bp were searched. MISA (Beier et al., 2017) was also used to identify simple sequence repeats. The minimum number of repeats was set to 10, 6, 5, 5, 5, and 5 for mononucleotide, dinucleotide, trinucleotide, tetranucleotide, pentanucleotide, and hexanucleotide, respectively.

### Phylogenetic Analyses

Phylogenetic analyses were performed using the Maximum Likelihood (ML) and Bayesian inference (BI) methods. For both ML and BI analyses, Jmodeltest v2.1.10 (Posada, 2008) was used to look for the best substitution model. The model GTR + GAMMA was chosen as the best model for ML and BI analyses. ML analysis was conducted in RAxML v8.2.12 (Stamatakis, 2014). The best ML tree was selected from 1,000 fast bootstrap replicates. BI analysis was conducted in MrBayes v3.2.7a (Ronquist et al., 2012). Markov Chain Monte Carlo (MCMC) runs were performed for 10 million generations with a sampling frequency of 1,000 generations. The temperature of the exchange chain was set to 0.2, and the “burninfrac” was set to 0.25, indicating that 2,500 burn-in samples were removed from the initial operation. The strict consequence tree and posterior probability (PP) were calculated from the remaining 7,500 trees. We chose *A. pacifica* as an outgroup for phylogenetic analyses because of its close relationship to the genus *Chrysanthemum* (Zhao et al., 2010; Liu et al., 2012; Ma et al., 2020).

### Phenotypic Analyses

After flower opening, flower traits were measured according to the handbook of UPOV (International Union for the Protection of New Varieties of Plants) and the DUS (Distinctness, Uniformity and Stability) test guidelines issued by the Ministry of Agriculture of the People’s Republic of China. Floral traits were classified according to four morphological indexes: inflorescence size, type of ray floret, type of flower head, and color of ray flowers. The inflorescence size was divided into two classes: large (>6 cm) and small (≤6 cm). The shape of the ray floret was divided into five types: ligulate, spatulate, quilled, incurved, and spoon-shaped. The flower head was divided into four types: without ray florets, single, semi-double, and double. Using visual analysis as in the UPOV and DUS guidelines, color of ray flowers was divided into eight categories: white, yellow, red, purple, pink, green, bicolor, and intermediate color.

## Results

### Characterization of cp Genomes

**Figure 2:**
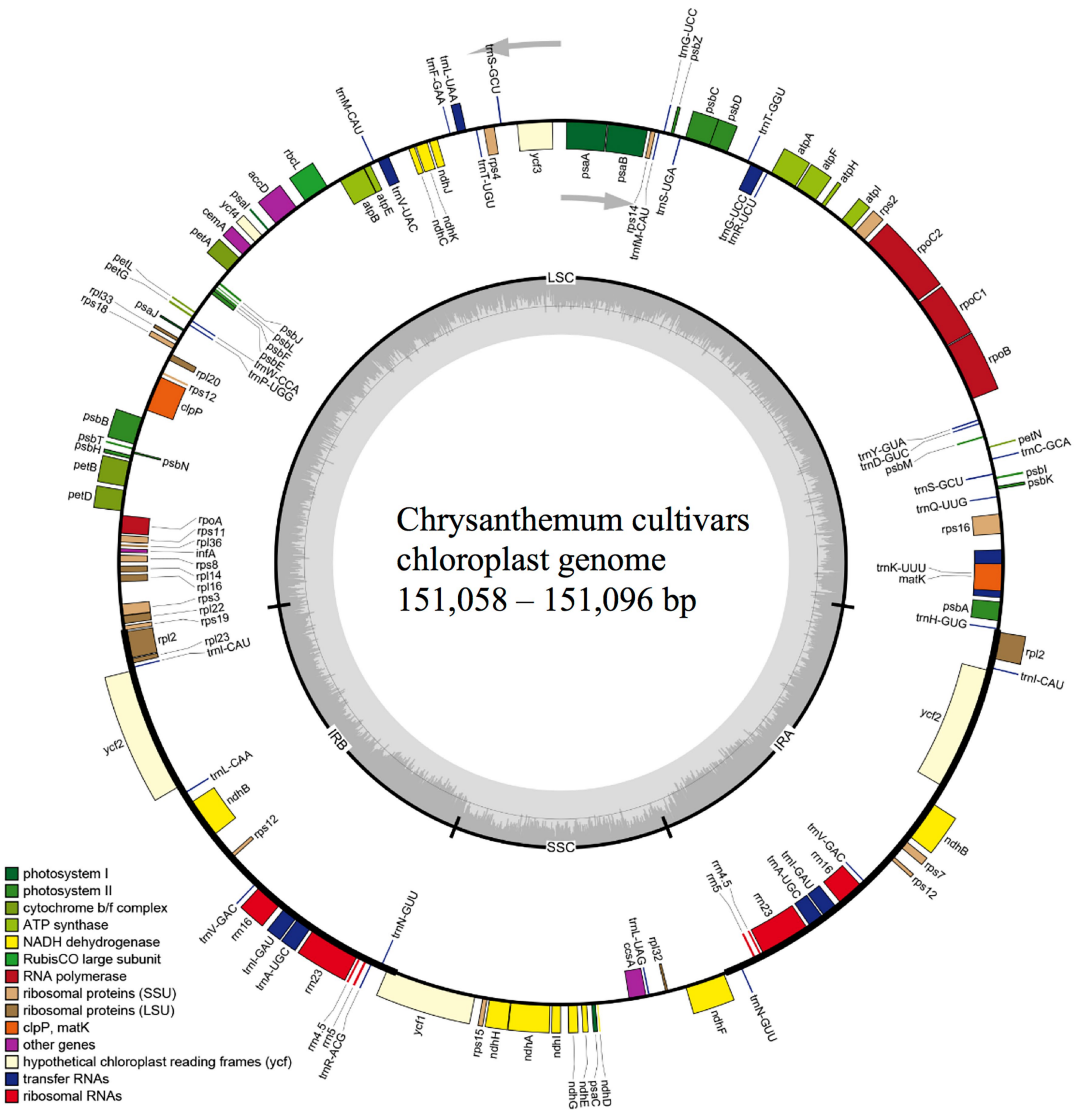
Chloroplast genome map of 36 chrysanthemum cultivars. Genes transcribed clockwise and counter-clockwise are showed inside and outside the circle, respectively. Genes belonging to different functional groups are color-coded. Dark gray in the inner circle corresponds to GC content. LSC, large single copy region; SSC, small single copy region; IRA and IRB, inverted repeats.

In this study, cp genomes of 36 cultivars of *C. morifolium* and the wild species, *C. nankingenese*, were assembled. The length, number of annotated genes, and GC content for each genome was presented in Supplementary Table S1. All cp genomes consisted of a large single copy region (LSC), a small single copy region (SSC), and two inverted repeats (IR; Figure 2). The total length of cp genomes ranged from 151,058 to 151,096 bp for the 36 cultivars, which included 82,856–82,858 bp LSC, 18,294 bp SSC, and 24,954–24,972 bp IR (Supplementary Table S1). The length of the *C. nankingenese* cp genome was 150,967 bp, which included 82,740 bp, 18,311 bp, and 24,958 bp of LSC, SSC, and IR regions, respectively (Supplementary Table S1). For all assembled cp genomes, 110 unique genes arranged in the same order were annotated, which consisted of 79 protein-coding genes, 27 tRNAs, and four rRNAs (Supplementary Table S1). The GC content of all cp genomes was 37.5% (Supplementary Table S1). The assembled cp genomes were deposited in GenBank (Table 1).

### Structure of cp Genomes

The distribution of genes in the IR and SC boundaries for the 36 *C. morifolium* cultivars and *C. nankingenese* was examined to explore the potential expansion and contraction of the IR boundary. The positions of IR and SC boundaries were conserved among the assembled cp genomes. The boundary between LSC and IRb was located within the *rps19* gene (Supplementary Figure S1). The length of the portions of the *rps19* gene in the LSC and IRb regions were 218 and 61 bp, respectively. The boundary between SSC and IRb occurred within the *ycf1* gene, with 558 and 4,457 bp (556 and 4,462 bp in *C. nankingenese*) of the gene located in the SSC and IRb regions, respectively (Supplementary Figure S1). The junction of SSC and IRa occurred 66 and 49 bp away from the *ndhF* gene (located in the SSC region) in the cp genomes of modern chrysanthemum cultivars and *C. nankingenese*, respectively (Supplementary Figure S1). The boundary between LSC and IRa was located between the *rpl*2 gene and the *trnH* gene. The *trnH* gene in the LSC region was 8 bp away from the boundary, while that of the *rpl*2 gene in IRa region was further away (Supplementary Figure S1). All assembled cp genomes were highly similar to the reference sequence (*C. indicum*) based on the results of mVISTA (Supplementary Figure S2). The coding regions were more conserved than the non-coding regions, and the most variable coding region was the *ycf1* gene (Supplementary Figure S2).

### Repeat Sequences of the cp Genome

We detected 43 dispersed repeats in the *C. morifolium* cultivar, “Fenban,” and 37 repeats in all the other cultivars (Supplementary Table S2). These repeats were either forward or palindromic. In *C. nankingenese*, 34 dispersed repeats, including 17 forward, 16 palindromic, and one complementary repeat, were identified (Supplementary Table S2). In addition to dispersed repeats, 41 SSRs in the cp genomes of *C. morifolium* cultivars, including 37 mononucleotide, one dinucleotide, and two trinucleotide SSRs, were detected (Supplementary Table S2). In *C. nankingenese*, 43 mononucleotides and two dinucleotide SSRs were found (Supplementary Table S2). There were no SSRs with a repeat unit longer than three nucleotides (e.g., tetranucleotide) in the cp genomes of *C. morifolium* cultivars and *C. nankingenese*.

### Phylogeny Reconstruction

Maximum Likelihood and BI analyses of cp genomes produced similar trees (Figure 1). The genus *Chrysanthemum* was monophyletic. All 36 cultivars of *C. morifolium* formed a strongly supported group (BS = 100, PP = 1.00), and wild species of the genus *Chrysanthemum* were paraphyletic to this group (Figure 1). The relationships among cultivars were largely unresolved because of low genetic diversity on cpDNA data (mean pair-wise diversity = 2.0 × 10^−6^), with the exception of five cultivars (“Cenluanbiran,” “Guohuaxingxinghuo,” “Jinjiliuxia,” “Nanshangaosi,” and “Stresa”) that shared a mutation and formed a clade with strong support (BS = 100, PP = 1.00) on the cpDNA tree (Figure 1). The wild species, *C. zawadskii*, was most closely related to the clade made up of the *C. morifolium* cultivars, followed by *Chrysanthemum lavandulifolium*, *Chrysanthemum chanetii*, and *Chrysanthemum boreale* (Figure 1). This result is different from a recent phylogenetic analyses based on cp genomes, in which the *C. lavandulifolium* was sister to the cultivars (Ma et al., 2020). This difference could be due to high cpDNA variation in *C. zawadskii*, and different chlorotypes have been sequenced in these two studies (Genbank accession number, MW539687 vs. MG799556). Three wild species, namely, *C. vestitum*, *C. indicum*, and *C. nankingenese* grouped together, with the latter two more closely related to each other.

### Phenotypic Characterization

The 36 *C. morifolium* cultivars showed very high morphological variation (Figure 1; Supplementary Figure S3; Table 1). All the Chinese traditional and the Japanese cultivars have large flowers and double flower heads; all the HD cultivars have small flowers and single (or semi-double) flower heads; and the Dutch cultivars have both large and small flowers, and three types of flower heads (single, semi-double, and double; Figure 1; Supplementary Figure S3; Table 1). There were four types of ray florets observed in the 36 cultivars (Figure 1; Supplementary Figure S3; Table 1). The ligulate and spoon types of ray florets were found in cultivars with large and small flowers, and ligulate was the most common type of ray floret (floret-type of 15 large flower and 10 small flower cultivars). The quilled and incurved types of ray floret were only found in four and one large flower cultivars, respectively. Flower color was successfully documented in 32 cultivars. The most common color was yellow (10 cultivars), followed by pink (6), purple (6), red (5), and white (3). The white and bicolors were rare and only produced by the “Cenluanbiran” and “Healing” cultivars, respectively.

## Discussion

### Maternal Genome Donor of Chrysanthemum Cultivars

Our phylogenetic analyses of cp genomes revealed that all chrysanthemum cultivars formed a strongly supported clade (BS = 100, BI = 1.00), and shared 64 substitutions that were distinct from wild species of the genus *Chrysanthemum* (Supplementary Data 1). This result is consistent with the suggestion that an extinct or un-sampled wild *Chrysanthemum* species served as the maternal donor of chrysanthemum cultivars (Ma et al., 2020; Qi et al., 2021). This conclusion, however, depends on the assumption that cpDNA variations of chrysanthemum cultivars and wild species have been well represented in phylogenetic analyses. Because there are more than 20,000 chrysanthemum cultivars all over the world, a comprehensive sampling with all cultivars is impossible. In this study, we collected 36 accessions to represent common cultivars of different countries and different flower morphologies. Our sample size is much larger than previous studies (12 accessions; Ma et al., 2020; Qi et al., 2021), and should comprise most, if not all, of the cpDNA variations in chrysanthemum cultivars. Domesticated chrysanthemum was thought to originate from multiple species, such as *C. argyrophyllum*, *C. indicum*, *C. lavandulifolium*, *C. nankingense*, *C. vestitum*, and *C. zawadskii* (Chen, 1985; Dai et al., 1998; Ma et al., 2020; Qi et al., 2021). A recent study revealed high cpDNA variations within two wild *Chrysanthemum* species, *C. indicum* and *C. vestitum* (Qi et al., 2021). Therefore, to elucidate whether the maternal parents of chrysanthemum cultivars were extinct or un-sampled, future studies should perform range-wild sampling of all wild species, and comparative analysis of whole cp genome sequences.

The genetic divergence between cultivars and wild species was slightly higher than that among wild species (0.0012 vs. 0.0010, *p* = 0.011, Wilcoxon rank sum test), which suggested that the maternal donor species diverged from the other wild species before the domestication of chrysanthemum. The divergent cp genomes of cultivars could also be explained by an increased rate of substitution caused by artificial selection for improving chrysanthemum ornamental value. However, in this case, we would expect that the high divergence between the cultivar lineages that experienced different artificial selection histories. In contrast, the divergence between cultivars was extremely low (mean pair-wise diversity = 2.0 × 10^−6^), regardless of their distinct floral morphologies. In addition, the chloroplast genome of plants is relatively conserved and contains genes involved in photosynthesis, transcription, and translation (Wicke et al., 2011), which were unlikely to be under directional selection during the breeding of *Chrysanthemum*.

### Utility of cp Genomes for Phylogenetic and Population Genetic Analyses

Chloroplast genomes are haploid, maternally inherited, and structurally conserved in most flowering plants, making the cp genome an ideal genetic markers for tracking the evolution of plants at both high and low taxonomic levels (Xing and Liu, 2008; Rogalski et al., 2015; Daniell et al., 2016). By taking advantage of next-generation sequencing technologies and bioinformatics tools, the cp genomes can be assembled from whole genome sequencing data, avoiding the time-consuming processes of chloroplast isolation and purification (Dierckxsens et al., 2017; Freudenthal et al., 2020; Jin et al., 2020). Cp genomes are widely used to study evolutionary history of plants, and provide a high resolution tool for deciphering phylogenetic relationships between closely related species, such as those in the families Asteraceae (Vargas et al., 2017), Fabaceae (Koenen et al., 2020), Fagaceae (Zhou et al.), Polemoniaceae (Rose et al., 2021), and Saxifragaceae (Folk et al., 2017). The phylogeny of the genus *Chrysanthemum* is well resolved based on the complete cp genomes from the current and a previous study (Ma et al., 2020), which contrasts with the low resolution phylogeny based on a few cpDNA fragments (Zhao et al., 2003; Qi et al., 2021).

Although single cpDNA fragments contain insufficient information to resolve the relationships for all species, concatenated analyses of multiple fragments may provide valuable information for species delimitation. Relatively high genetic variation in the intergenic (e.g., *trnH*-*psbA*) and the coding regions of *ycf*1 was found. Some of these regions are used as genetic markers for phylogenetic analyses in land plants (CBOL Plant Working Group, 2009; Dong et al., 2015; Bagheri et al., 2017), and may be promising DNA-barcode markers in the genus *Chrysanthemum*. Simple repeats in the cp genome (i.e., cpSSRs) are important genetic markers for population genetic analyses. The mutation rate in cpSSR regions has been estimated at 3.2 × 10^−5^–7.9 × 10^−5^ per site per year (Provan et al., 1999), thousands of times higher than elsewhere in the cp genome (1 × 10^−9^–3 × 10^−9^ per site per year; Wolfe et al., 1987). Thus, the cpSSRs found in this study have the potential to be used to detect genetic variations between recently diverged lineages, such as chrysanthemum cultivars or populations of wild chrysanthemum species. However, it worth noting that sequencing repetitive regions is technically challenging for next-generation sequencing (Treangen and Salzberg, 2011). New laboratory methods and novel computational tools will be required to accurately genotype the cpSSR regions in plants (Šarhanová et al., 2018).

## Conclusion

We assembled 37 cp genomes of chrysanthemum cultivars and wild species. The structure of these cp genomes was highly conserved in the genus *Chrysanthemum*, with similar IR-SSC boundaries, number and order of genes, and content of repetitive elements. Phylogenetic analyses based on cpDNA data revealed a strongly supported clade formed by chrysanthemum cultivars, suggesting a lineage of the genus *Chrysanthemum* as well as its subsequent cultivars unidirectionally providing a maternal source in breeding programs for developing modern cultivars. The high divergence between the cp genomes of chrysanthemum cultivars and wild species indicates that the maternal parent might be an extinct or un-sampled species (or population). Moreover, the low cpDNA polymorphism in chrysanthemum cultivars suggests that either the maternal parent had very low cpDNA variation, or only a few individuals served as the maternal donor of modern cultivars. In addition to maternal origin information of chrysanthemum cultivars, this study provides cp genomic resources for developing genetic markers that can be used in phylogenetic and phylogeographic studies of the genus *Chrysanthemum*. Specially, we suggest that repetitive regions (e.g., cpSSR) with elevated mutation rates may contain enough genetic variation be used to delineate chrysanthemum cultivars and populations of wild relatives. This study sheds new light on the origin of chrysanthemum cultivars and provides a valuable genetic resource for the continued development of varieties.

## Data Availability Statement

The data present in the study are deposited in the Genbank (https://www.ncbi.nlm.nih.gov/genbank/), accession number ON534022-ON534058.

## Author Contributions

HX and BW designed this study. YX, BL, and HX collected samples. YX and BL analyzed the data. HX, BW, and KO wrote the original draft, HZ, FW, and HX supervised the project. All authors read and approved the final manuscript.

## Funding

This work was supported by the Major Technological Innovation of Guangdong Province of China (no. 2020B020220009) and the National Natural Science Foundation of China (no. 32001244).

## Conflict of Interest

The authors declare that the research was conducted in the absence of any commercial or financial relationships that could be construed as a potential conflict of interest.

## Publisher’s Note

All claims expressed in this article are solely those of the authors and do not necessarily represent those of their affiliated organizations, or those of the publisher, the editors and the reviewers. Any product that may be evaluated in this article, or claim that may be made by its manufacturer, is not guaranteed or endorsed by the publisher.

## Footnotes

^1^ https://ncbiinsights.ncbi.nlm.nih.gov/tag/sequin/
