# Supplementary material for: The Maternal Donor of Chrysanthemum Cultivars Revealed by Comparative Analysis of the Chloroplast Genome
Source: Front Plant Sci. 2022 Jun 2;13:923442. doi: 10.3389/fpls.2022.923442 (PMC9202620; doi:10.3389/fpls.2022.923442)
Supplement: Supplementary file 1 [file Data_Sheet_1.PDF]

Table S1 Characterization of the 37 chrysanthemum chloroplast genomes assembled in this study.

Sample	Length(bp) Total	LSC	SSC	IR	Number of genes (unique) Total	PCG	tRNA	rRNA	GC content (%)
**Cultivar**									
Cenluanbiran	151,060	82,858	18,294	24,954	125(110)	83(79)	34(27)	8 (4)	37.5
Caixuechuntao	151,060	82,858	18,294	24,954	125(110)	83(79)	34(27)	8 (4)	37.5
Casa	151,060	82,858	18,294	24,954	125(110)	83(79)	34(27)	8 (4)	37.5
Donghainijin	151,060	82,858	18,294	24,954	125(110)	83(79)	34(27)	8 (4)	37.5
Donghaishenyun	151,060	82,858	18,294	24,954	125(110)	83(79)	34(27)	8 (4)	37.5
Fenban	151,096	82,858	18,294	24,972	125(110)	83(79)	34(27)	8 (4)	37.5
Feiyunjuanshen	151,060	82,858	18,294	24,954	125(110)	83(79)	34(27)	8 (4)	37.5
Guohuacai	151,060	82,858	18,294	24,954	125(110)	83(79)	34(27)	8 (4)	37.5
Guohuafentao	151,060	82,858	18,294	24,954	125(110)	83(79)	34(27)	8 (4)	37.5
Guohuaxingxinghuo	151,060	82,858	18,294	24,954	125(110)	83(79)	34(27)	8 (4)	37.5
Guohuayulaiguang	151,060	82,858	18,294	24,954	125(110)	83(79)	34(27)	8 (4)	37.5
Gusifoguang	151,058	82,856	18,294	24,954	125(110)	83(79)	34(27)	8 (4)	37.5
Gustavoorange	151,060	82,858	18,294	24,954	125(110)	83(79)	34(27)	8 (4)	37.5
Avron	151,060	82,858	18,294	24,954	125(110)	83(79)	34(27)	8 (4)	37.5
Bonbonyellow	151,060	82,858	18,294	24,954	125(110)	83(79)	34(27)	8 (4)	37.5
Junhebaiyun	151,060	82,858	18,294	24,954	125(110)	83(79)	34(27)	8 (4)	37.5
Jinjiliuxia	151,060	82,858	18,294	24,954	125(110)	83(79)	34(27)	8 (4)	37.5
Florange	151,060	82,858	18,294	24,954	125(110)	83(79)	34(27)	8 (4)	37.5
Jingxingzhicheng	151,060	82,858	18,294	24,954	125(110)	83(79)	34(27)	8 (4)	37.5
Stresa	151,060	82,858	18,294	24,954	125(110)	83(79)	34(27)	8 (4)	37.5
Healing	151,060	82,858	18,294	24,954	125(110)	83(79)	34(27)	8 (4)	37.5
Matisse	151,060	82,858	18,294	24,954	125(110)	83(79)	34(27)	8 (4)	37.5
Nanshangaosi	151,058	82,856	18,294	24,954	125(110)	83(79)	34(27)	8 (4)	37.5
Panlongjiangcheng	151,060	82,858	18,294	24,954	125(110)	83(79)	34(27)	8 (4)	37.5
Radostyellow	151,060	82,858	18,294	24,954	125(110)	83(79)	34(27)	8 (4)	37.5
Jinba	151,060	82,858	18,294	24,954	125(110)	83(79)	34(27)	8 (4)	37.5
Taipingbao	151,060	82,858	18,294	24,954	125(110)	83(79)	34(27)	8 (4)	37.5
Taipinghonglian	151,060	82,858	18,294	24,954	125(110)	83(79)	34(27)	8 (4)	37.5
Mundoorange	151,060	82,858	18,294	24,954	125(110)	83(79)	34(27)	8 (4)	37.5
Zhenziju	151,060	82,858	18,294	24,954	125(110)	83(79)	34(27)	8 (4)	37.5
Yunshandiezi	151,060	82,858	18,294	24,954	125(110)	83(79)	34(27)	8 (4)	37.5
Xinxinghuo	151,060	82,858	18,294	24,954	125(110)	83(79)	34(27)	8 (4)	37.5
Ziban	151,060	82,858	18,294	24,954	125(110)	83(79)	34(27)	8 (4)	37.5
Zihongtuogui	151,060	82,858	18,294	24,954	125(110)	83(79)	34(27)	8 (4)	37.5
Zilongtanzhua	151,060	82,858	18,294	24,954	125(110)	83(79)	34(27)	8 (4)	37.5
Ziyan	151,060	82,858	18,294	24,954	125(110)	83(79)	34(27)	8 (4)	37.5
**Wild speceis**									
C. nankingenese	150,967	82,740	18,311	24,958	125(110)	83(79)	34(27)	8 (4)	37.5

Table S2 Number and type of repeats in chloroplast genomes of chrysanthemum cultivars and their wild relatives.

Sample	Forward	Reverse	Complement	Palindromic	Total
**Cultivar**					
Cenluanbiran	19	0	0	18	37
Caixuechuntao	19	0	0	18	37
Casa	19	0	0	18	37
Donghainijin	19	0	0	18	37
Donghaishenyun	19	0	0	18	37
Fenban	22	0	0	21	43
Feiyunjuanshen	19	0	0	18	37
Guohuacai	19	0	0	18	37
Guohuafentao	19	0	0	18	37
Guohuaxingxingh	19	0	0	18	37
Guohuayulaiguan	19	0	0	18	37
Gusifoguang	19	0	0	18	37
Gustavoorange	19	0	0	18	37
Avron	19	0	0	18	37
Bonbonyellow	19	0	0	18	37
Junhebaiyun	19	0	0	18	37
Jinjiliuxia	19	0	0	18	37
Florange	19	0	0	18	37
Jingxingzhicheng	19	0	0	18	37
Stresa	19	0	0	18	37
Healing	19	0	0	18	37
Matisse	19	0	0	18	37
Nanshangaosi	19	0	0	18	37
Panlongjiangchen	19	0	0	18	37
Radostyellow	19	0	0	18	37
Jinba	19	0	0	18	37
Taipingbao	19	0	0	18	37
Taipinghonglian	19	0	0	18	37
Mundoorange	19	0	0	18	37
Zhenziju	19	0	0	18	37
Yunshandiezi	19	0	0	18	37
Xinxinghuo	19	0	0	18	37
Ziban	19	0	0	18	37
Zihongtuogui	19	0	0	18	37
Zilongtanzhua	19	0	0	18	37
Ziyan	19	0	0	18	37
**Wild speceis**					
C. nankingenese	17	0	1	16	34

Table S3 Number and type of simple sequence repeat (SSR) in chloroplast genomes of chrysanthemum cultivars and their wild relatives.

Sample	Mononucleotide	Dinucleotide	Trinucleotide	Total
**Cultivar**				
Cenluanbiran	38	1	2	41
Caixuechuntao	38	1	2	41
Casa	38	1	2	41
Donghainijin	38	1	2	41
Donghaishenyun	38	1	2	41
Fenban	38	1	2	41
Feiyunjuanshen	38	1	2	41
Guohuacai	38	1	2	41
Guohuafentao	38	1	2	41
Guohuaxingxinghuo	38	1	2	41
Guohuayulaiguang	38	1	2	41
Gusifoguang	38	1	2	41
Gustavoorange	38	1	2	41
Avron	38	1	2	41
Bonbonyellow	38	1	2	41
Junhebaiyun	38	1	2	41
Jinjiliuxia	38	1	2	41
Florange	38	1	2	41
Jingxingzhicheng	38	1	2	41
Stresa	38	1	2	41
Healing	38	1	2	41
Matisse	38	1	2	41
Nanshangaosi	38	1	2	41
Panlongjiangcheng	38	1	2	41
Radostyellow	38	1	2	41
Jinba	38	1	2	41
Taipingbao	38	1	2	41
Taipinghonglian	38	1	2	41
Mundoorange	38	1	2	41
Zhenziju	38	1	2	41
Yunshandiezi	38	1	2	41
Xinxinghuo	38	1	2	41
Ziban	38	1	2	41
Zihongtuogui	38	1	2	41
Zilongtanzhua	38	1	2	41
Ziyan	38	1	2	41
**Wild speceis**				
C. nankingenese	43	2	0	45

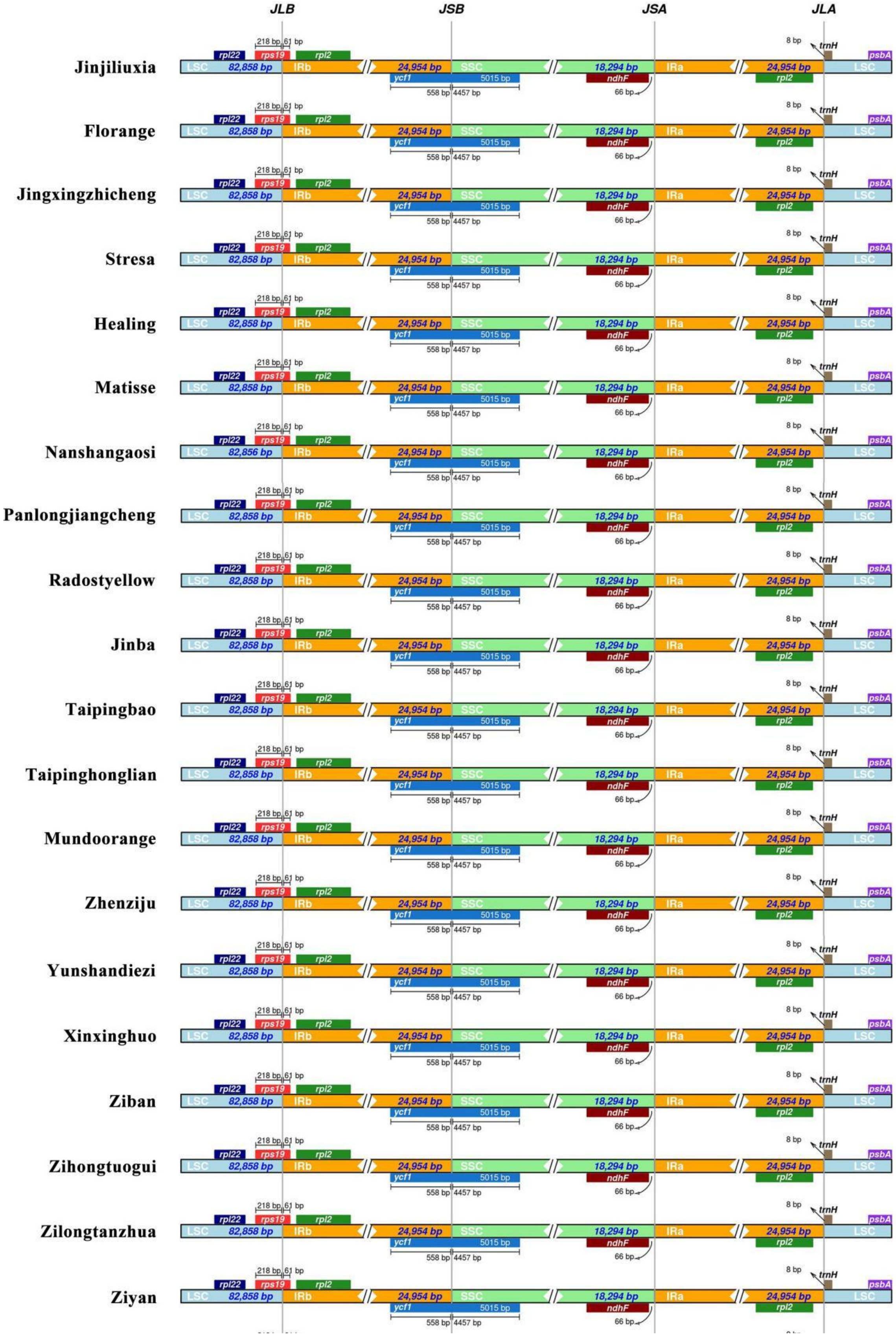
JLB
JSB
JSA
JLA
Jinjiliuxia
Florange
Jingxingzhicheng
Stresa
Healing
Matisse
Nanshangaosi
Panlongjiangcheng
Radostyellow
Jinba
Taipingbao
Taipinghonglian
Mundoorange
Zhenziju
Yunshandiezi
Xinxinghuo
Ziban
Zihongtuogui
Zilongtanzhua
Ziyan
218 bp
61 bp
rpl22
rps19
rpl2
LSC
82,858 bp
82,856 bp
IRb
24,954 bp
SSC
ycf1
5015 bp
558 bp
4457 bp
18,294 bp
ndhF
66 bp
IRa
8 bp
trnH
psbA

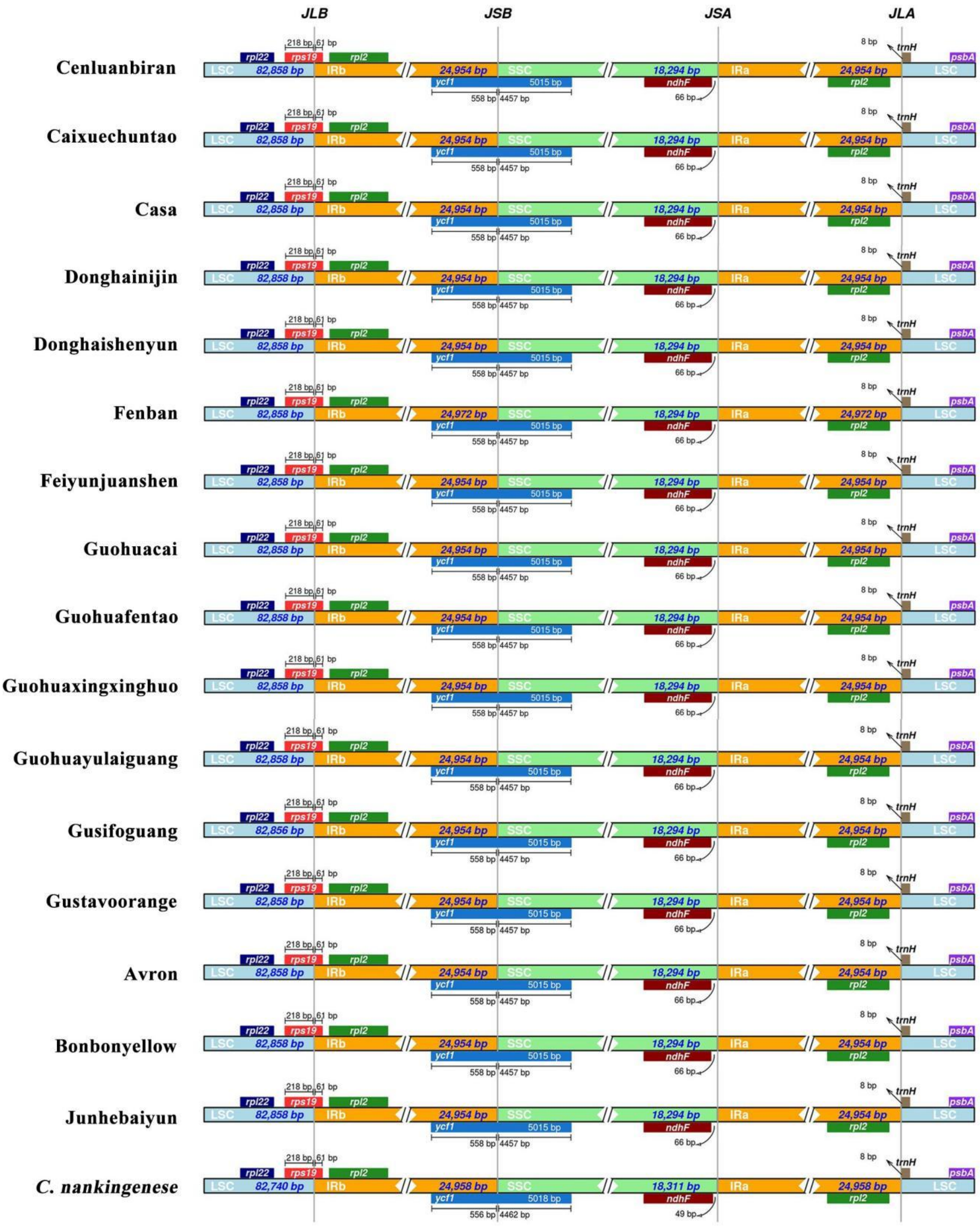

Fig. S1 The distribution of genes in the IR and SC boundary regions for the 36 C. morifolium cultivars and C. nankingenese. The figure is not to scale with respect to sequence length and only shows relative changes at or near the IR/SC borders. JLB, Junction of LSC and IRb; JSB, Junction of SSC and IRb; JSA, Junction of SSC and IRa; JLA, Junction of SSC and IRa.

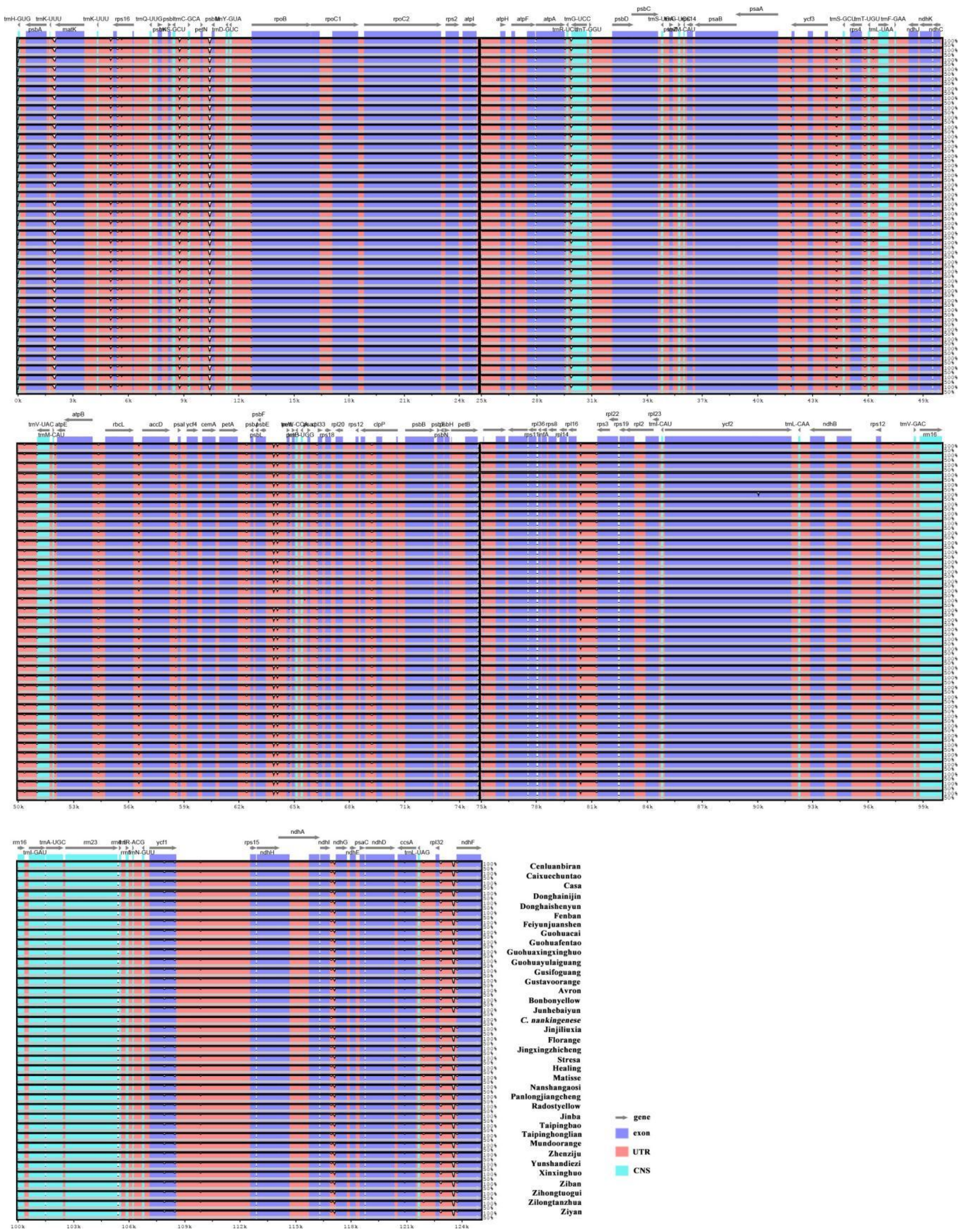
Cenluanbiran
Caixuechuntao
Casa
Donghainijin
Donghaishenyun
Fenban
Feiyunjuanshen
Guohuacai
Guohuafentao
Guohuaxingxinghuo
Guohuayulaiguang
Gusifoguang
Gustavoorange
Avron
Bonbonyellow
Junhebaiyun
C. nankingenese
Jinjiliuxia
Florange
Jingxingzhicheng
Stresa
Healing
Matisse
Nanshangaosi
Panlongjiangcheng
Radostyellow
Jinba
Taipingbao
Taipinghonglian
Mundoorange
Zhenziju
Yunshandiezi
Xinxinghuo
Ziban
Zihongtuogui
Zilongtanzhua
Ziyan
gene
exon
UTR
CNS

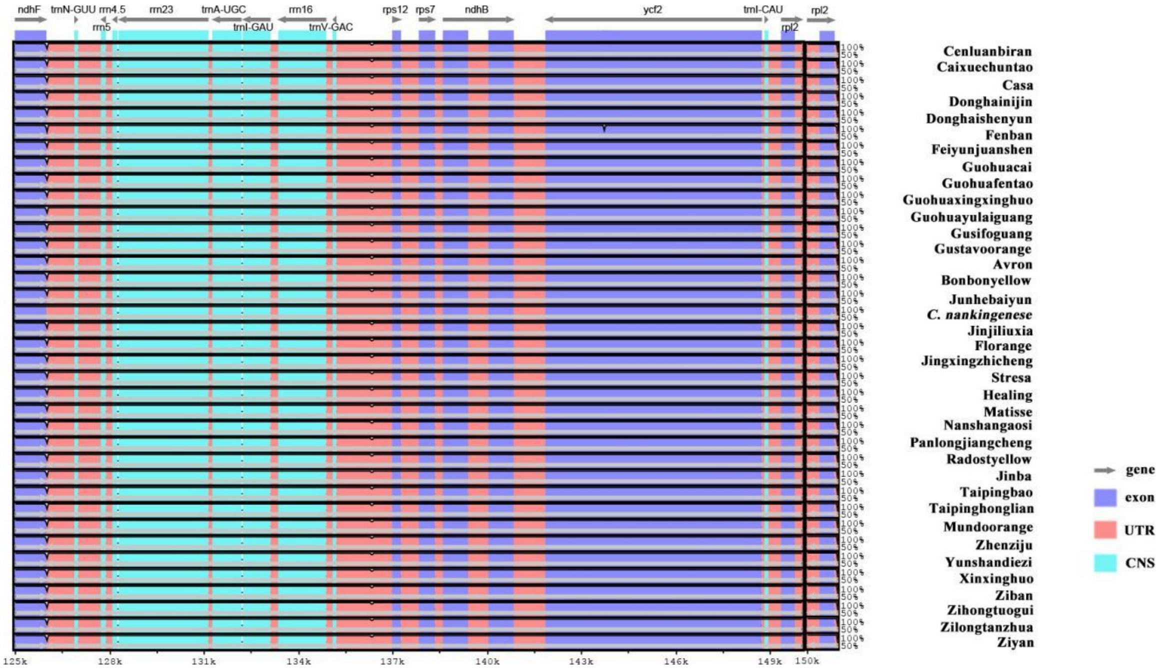

Fig. S2 Alignment of chloroplast genomes among 36 C. morifolium cultivars and their wild relatives.

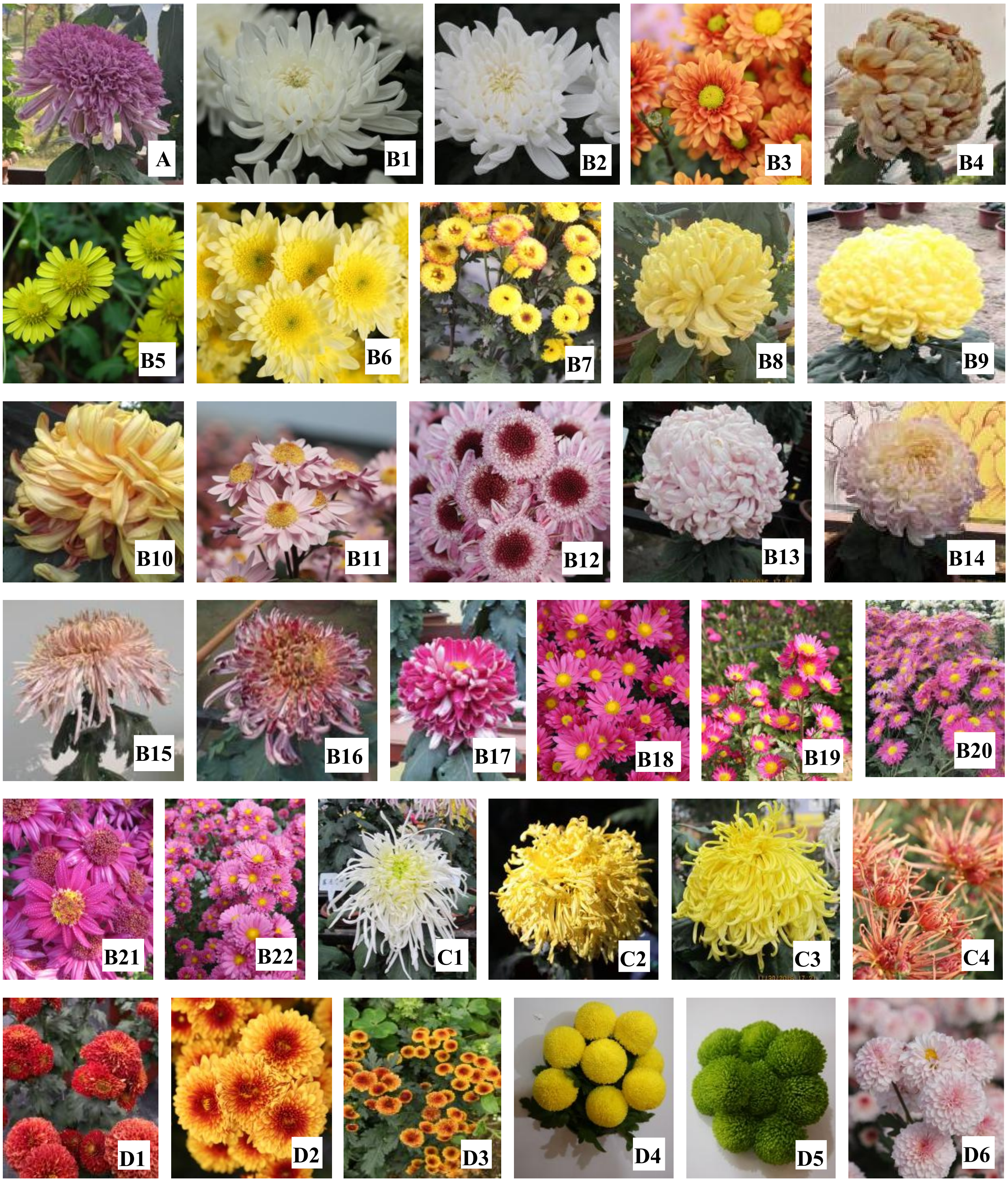

Fig. S3 Flower morphologies of representative chrysanthemum cultivars. A, Zilongtanzhua; B1, Jinba; B2, Jingxingzhicheng; B3, Florange; B4, Donghainijin; B5, *Chrysanthemum nankingenese*; B6, Radostyellow; B7, Casa; B8, Gusifoguang; B9, Guohuayulaiguang; B10, Taipinghonglian; B11, Fenban; B12, Stresa; B13, Caixuechuntao; B14, Panlongjiangcheng; B15, Feiyunjuanshen; B16, Donghaishenyun; B17, Cenluanbiran; B18, Zhenziju; B19, Ziyan; B20, Ziban; B21, Zihongtuogui; B22, Yunshandiezi; C1, Taipingbao; C2, Jinjiliuxia; C3, Nanshangaosi; C4, Xinxinghuo; D1, Avron; D2, Gustavoorange; D3, Mundoorange; D4, Bonbonyellow; D5, Healing; D6, Matisse. A, Incurved; B1-B22, Ligulate; C1-C4, Quilled; D1-D6, Spoon.
